# English law for the surgeon II: Clinical negligence

**DOI:** 10.1186/1758-3284-3-52

**Published:** 2011-12-21

**Authors:** Waseem Jerjes, Jaspal Mahil, Tahwinder Upile

**Affiliations:** 1 UCL Department of Surgery, University College London Medical School, London, UK; 2 Leeds Institute of Molecular Medicine, School of Medicine, University of Leeds, Leeds, UK; 3 Department of Otolaryngology/Head and Neck Surgery, Barnet and Chase Farm hospitals NHS Trust, Enfield, UK; 4 UCLH Head and Neck Unit, London, UK

## Abstract

Traditionally, in the United Kingdom and Europe, the surgeon was generally not troubled by litigation from patients presenting as elective as well as emergency cases, but this aspect of custom has changed. Litigation by patients now significantly affects surgical practice and vicarious liability often affects hospitals. We discuss some fundamental legal definitions, a must to know for a surgeon, and highlight some interesting cases.

## Review

Expert medical opinion is essential in helping the courts to assess whether or not the surgeon has exercised a standard level of care under the circumstances. Depending on the testimony of those professionals, the court should decide if the defendant achieved that standard; in other words: negligent or not [1]. Of course the defendant surgeon standard of care will be compared to the standard of care in the same surgical discipline [2].

### Civil law and clinical negligence

The civil law of negligence is designed to provide compensation for one individual injured by another's negligence. Negligence can be determined by a threefold test. Initially, a duty of care must be established, a breach to that duty has occurred and this has caused harm to that person [3]. In a surgical setting, it is automatically assumed that the examining or the operating surgeon has a duty of care; difficulty arises where a person has not been accepted as a patient. English law does not oblige anyone to be a Good Samaritan [4]. When that standard of care provided by that surgeon falls below the standard of a "responsible body of medical/surgical men" and leads to harm to the patient, here the surgeon is found negligent [5].

The "custom test" is usually applied to assess the surgeon's standard of care. A surgeon is considered to have deviated from normal practice if it was proved that there is a usual and normal practice, this practice was not adopted by the surgeon and the surgeon adopted a practice in which no professional man of ordinary skills would have taken [6].

Historically, negligence was recognised as part of the torts until 1932. *Donoghue v Stevenson *was related to the claims of the decomposed remnants of a snail in a ginger beer bottle. Since then a "duty of care" was established; in this case, the duty of the manufacturers of foods and drinks towards the public [7]. Soon this has been applied in every single field including surgery.

During the last half a century, *Bolam v Friern Hospital Management Committee *laid down the rule when assessing the standard of care in negligence and has been quoted in almost every clinical negligence case in England and Wales.

In 1954, John Hector Bolam who suffered from clinical depression agreed to be admitted to a mental health institution run by Friern Hospital Management Committee to undergo electro-convulsive therapy. The procedure involved initiating a number of medically controlled seizures. Prior to the procedure, no relaxant drugs were administered nor were restraints used. Unfortunately, Mr Bolam flailed about violently and suffered multiple injuries including hip fracture.

Eventually, Mr Bolam started proceedings against Friern Hospital Management Committee accusing them of negligence for not administering the relaxants, not restraining him and failing to provide fully informed consent as he was never told about the risks involved with such therapy.

During the proceedings, expert witnesses had confirmed that the majority of the medical opinions are not in favour of using relaxants and applying restraints as these could actually increase the risks of complications including fractures. The expert witnesses also elaborated that, at that time, it is not a common practice to warn the patients about small risk of treatment.

During that period, juries were being used for tort cases in England and Wales and it was their duty to hold the defendant liable or not. The jury delivered a verdict in favour of the defendant hospital. Mr Justice McNair said "I myself would prefer to put it this way, that he is not guilty of negligence if he has acted in accordance with a practice accepted as proper by a responsible body of medical men skilled in that particular art...At the same time, that does not mean that a medical man can obstinately and pig-headedly carry on with some old technique if it has been proved to be contrary to what is really substantially the whole of informed medical opinion". The *locus classicus *of the test for the standard of care developed from this landmark.

### The *Bolam *principle

When there are two or more conflicting surgical opinions with regard to diagnosing or treating a patient, the Bolam principle is applied. The technical issues involved with the Bolam principle makes it difficult for the courts when dealing with negligence compared to non-medical cases or cases that does not involve healthcare professionals. Although a duty of care can be easily established but setting the standard of care is very difficult. It is not difficult to find two surgeons disagree on the optimal treatment offered to a single patient. Also, patients arrive to hospital complaining of symptoms; if developed complications post surgery, it would be difficult to establish if it is due to substandard care or just a sequel of their pre-existing symptoms and hence establish the connection between breach of care and harm caused by that breach (i.e. a patient suffering from sensory and/or motor weakness as a result of a fracture; when the fracture is fixed and the symptoms persist, no one can tell if the symptoms are the result from the fracture injury or a post surgical complication). Also, when an intervention or non-intervention is applied and it was found not to improve the prognosis, would the surgeon be hold liable if offered no care? (i.e. a surgeon not performing a laparotomy on a complicated patient suffering from perforation).

In 1964, the bankers an advertising partnership, Hedley Byrne, was about to undertake some significant advertising contracts for Easipower Ltd., and wanted to be sure of their financial security [8]. The bankers telephoned the bank of Heller & Partners Ltd. inquiring about the financial state and credit record of their client. Heller vouched for their client's record but qualified it by waiving responsibility, stating that the information was: "for your private use and without responsibility on the part of the bank and its officials." Hedley Byrne relied on that information and entered into a contract with Easipower which went bankrupt soon afterwards. Hedley Byrne sued Heller for negligence. The court found that the relationship between the parties was "sufficiently proximate" as to create a duty of care. Here a reasonable reliance is created and have been applied, alongside with Bolam, to assess negligence in advisory activities (i.e. diagnosis, treatment and prognosis).

In *Akenzua v Secretary of State for the Home Department *[9], the police were held liable when they released a dangerous criminal, to act as an informant, who was due to be deported. This resulted in the death of a member of the public. The police had a duty of care towards the public and given the criminal's record, the officials must at least have been reckless and exercising that power must have known that it was illegal. It was enough that it was foreseeable that the criminal would harm somebody. In a similar case, *Palmer v Tees Health Authority*, a doctor was accused of negligence to diagnose that there was a real, substantial, and foreseeable risk of a psychiatric out-patient committing serious sexual offences against children; this has lead to the murder of a four-year-old child. The court struck out the claim on the grounds that there was no duty of care towards the child, as any child, at any time, was in the same danger. Furthermore, as the patient did not suffer from a treatable mental illness, there was no legal right to either treat or detain the person [10].

A woman developed an abscess after she had her ear pierced by a jeweler. Subsequently she sued for negligence as the instruments used were not aseptically sterile. However, the standard of care provided by that jeweler was the same as any other jeweler. Thus, no compensation was given; the woman expected a higher standard of care that may be provided by a surgeon not a jeweler [11].

In West Midlands Regional Health Authority, 2 consultants (physician and surgeon) instead of waiting for sputum results for a patient who presented with symptoms of tuberculosis, carried out an endoscopic diagnostic surgery to rule out other diseases including cancer. The patient, who was subsequently diagnosed with tuberculosis, suffered paralysis of the left vocal cords following damage to the left recurrent laryngeal nerve. The decision of the physician and the surgeon to proceed was said by their expert peers to be reasonable in the circumstances [12].

A casualty officer in Chelsea & Kensington Hospital, being unwell and failing to attend, gave instructions to discharge three patients who attended earlier to the emergency department. One patient died, later, due to arsenical poisoning, a rare cause of death. The hospital was found negligent in failing to examine the patient but not the death of that patient [13]. The court was satisfied that even if the defendants had performed their duty of care and admitted the deceased to their hospital, he would still have died of arsenic poisoning five hours after being admitted, and that he therefore suffered no loss as a consequence of the breach of duty complained of [14].

When an overtired or overworked surgeon has mistaken a diagnosis or commits a surgical error, he/she is liable to the injured patient. The lack of resources often causes surgeons to work for excessive continuous hours and in one judgment, a judge said "the lack of resources should be taken into account in medical negligence claims" [15]. But this was later rejected in *Brooks v Home Office *[16].

In another case, a mother in a high-risk pregnancy had been in labour for 22 hours. The senior registrar applied forceps to assist the delivery. The newborn suffered severe brain damage. The surgeon was not found negligent as the standard of care did not fall below that of a reasonable doctor in the circumstances [17].

A neurosurgeon was not found negligent when he failed to inform the patient of a very low risk (1%) of paraplegia following cervical cord de-compression in which the patient subsequently underwent and suffered this rare complication [18]. It was argued that the doctor doesn't need to provide information about possible remote side effects. Lord Scarman stated "I have to say that a judge's 'preference' for one body of distinguished professional opinion to another also professionally distinguished is not sufficient to establish negligence in a practitioner whose actions have received the seal of approval of those whose opinions, truthfully expressed, honestly held, were not preferred". This provided strong endorsement to the Bolam principle. Also, although the court might prefer one body of surgical opinion to other, but this doesn't mean that a surgeon who adopted that opinion can found to be negligent.

On a balance of probability, a judge has rejected the appeal in *Hotson v East Berkshire Area Health Authority *[19]. A 20-year-old and after failing to diagnose the extent of his hip injuries 7 years ago, exhibited deformity of the hip joint and permanent disability. The case was rejected on the grounds that even if the diagnosis had been made correctly, there was still a 75% risk of the plaintiff's disability developing, but that the medical staff's breach of duty had turned that risk into an inevitability, thereby denying the plaintiff a 25% chance of a good recovery, and hence only 25% of the full values of damage were granted.

In *Wilsher v Essex Area Health Authority *[20], a junior doctor administered excessive oxygen to a premature child during the post-natal care; this lead to blindness. The medical experts provided evidence that there are five further causes that might have lead to blindness. The Lords, therefore, found that it was impossible to say that it had caused, or materially contributed, to the injury and the claim was dismissed.

When it comes to trainees, the courts make no allowance when assessing liability. In a number of cases courts has rejected that junior doctors are inexperienced and mistakes can happen. According to the courts, junior doctors are required to adhere to the same standard of care as their senior colleagues; and also seek senior opinion when required. At that stage the term "team negligence" was introduced. In *Jones v Manchester Corporation *[21], the Court of Appeal did not accept the evidence that the inexperience of the doctor has lead him to administer excessive dose of anaesthesia which lead to the patient's death. One point was made clear in this case, a junior doctor, who, recognising his inexperience, calls in his consultant will have discharged his duty. Responsibility in the law will move to the consultant.

When it comes to continuous professional development, a doctor is expected to be up to date with all the recent developments in the field, also need to be aware of all the abandoned techniques or therapies. However, where to draw the line, is always debatable. A doctor was found guilty when a patient developed brachial palsy following blood transfusion. The evidence was based on a publication in a prominent journal 6 months previously. It was an interesting case where a judgment was based on a published article. Wisely, the Court of Appeal overturned the case suggesting that it would be impossible to expect every doctor to read every single published article [22]. It used to be acceptable when a surgeon says that his/her current surgical practice was the acceptable practice when he/she started the training 10-20 years ago, for example. Nowadays this surgeon can be found negligent if its current practice is not up to date.

A patient in the Royal Prince Alfred Hospital who had been born with a spinal problem had her spinal cord totally severed, following the operation, leaving her paraplegic. Mr. Justice Reynolds found that a major issue was the relationship between the hospital and the surgeons, that the hospital was not liable or vicariously liable but that the surgeons who performed the operation were negligent [23].

The principle of the so-called "super-specialist" was well protected by the Bolam principle when a spinal surgeon was not found negligent after operating on a patient who eventually sustained paraplegia. The majority of the evidence provided by his colleagues confirm that the operation represent high-risk and has been abandoned. Only 11 surgeons in the whole country were using such approach [24].

### The *Bolitho *principle

Few attempts were employed by the courts to move away from the Bolam principle (or test) but in almost every case higher courts have overruled their appeals. In *Bolitho v City and Hackney Health Authority*, the claim relates to treatment received by Patrick Nigel Bolitho at St. Bartholomew's Hospital on 1984 when he was two years old. Patrick suffered catastrophic brain damage as a result of the bronchial air passages becoming blocked leading to cardiac arrest. The doctor who, negligently, failed to attend said that she would not have intubated the boy even if she would have attended as she weighted risks against benefits; at that time it was agreed that the only course of action to prevent the damage was to have the boy intubated. Six expert witnesses were brought to testify, in which five said they would have carried the procedure and one disagreed. By the end of the trial it was common ground, first, that intubation so as to provide an airway in any event would have ensured that the respiratory failure which occurred did not lead to cardiac arrest and, second, that such intubation would have had to be carried out, if at all, before the final catastrophic episode [25].

The House of Lords held that there would have to be a logical basis for the opinion not to intubate. This means that a judge will be entitled to choose between two bodies of expert opinion and to reject an opinion which is 'logically indefensible'. This has been interpreted as being a situation where the court sets the law for clinical negligence.

Sometimes certain conduct might add to the risk of injury, if the defendant engages in such conduct in breach of a common law duty, and if the injury is the kind to which the conduct related, then the defendant is taken to have caused the injury even though the existence and extent of the contribution made by the breach cannot be ascertained. In *McGhee v National Coal Board *[26], the House of Lords held that where a breach of duty has a material effect on the likelihood of injury then the subsequent injury will be said to have been caused by the breach. McGhee, who was employed to clean out brick kilns, sued his employer for negligence for failing to provide the proper washing facilities to prevent the outbreak of dermatitis from the accumulation of coal dust on his skin. The issue before the House of Lords was whether the failure to provide the washing facilities had caused the rash. Lord Reid stated: "The medical evidence is to the effect that the fact that the man had to cycle home caked with grime and sweat added materially to the risk".

## Discussion

The Bolam principle considered until now to be a good screening modality for standard care. Practice in surgery has not reached the stage of scientific reliability where answer can be given. With Bolam, a test can be validated when two or more genuine and respected reviews are held, as the standard of care is a matter of medical judgment. However academics believe that it seems to overprotect surgeons and also allows them to set their own standards.

The main criticism to the Bolam principle as a descriptive test of what is actually done, while negligence cases usually on what should be done. The definition of a competent body of opinion is imprecise and can legitimize marginal practices. Solicitors often find it to be inconsistent with negligence principles generally.

The most essential component in negligence is to prove that the surgeon failed under the circumstances to provide an acceptable standard care to his/her patient. The circumstances vary in every single case and it is almost impossible to compare two cases; also every surgeon would react differently to the circumstances depending on the level of experience, knowledge, training and school of thought. The Bolam principle is one way to show that the care provided was standard or substandard. The Bolam principle has been perceived as being reliant on the testimony of the professionals supporting the defendant. The Bolitho case by implication imposes that the standard proclaimed must be justified on a logical basis taking into consideration the risks and benefits. The shift from the Bolam to the Bolitho approach allowed the court to reach its own conclusions based on logic rather than knowledge after taking more enquiring stance to test the medical evidence offered by both parties in litigation.

In *Penney v East Kent Health Authority*, three women developed cervical cancer after a negative screening test. Despite that the claimants' slides showed abnormal cells, they were labeled as normal. The health authority defence was that these findings (abnormal cells) are differently interpreted and this should be decided according to the Bolam test. The trial judge and the Court of Appeal agreed that the "logical" act by a reasonable person is to label these slides as borderline so Bolitho was preferred over Bolam [27].

It was felt that judges used to be reluctant to find surgeons guilty of negligence and many judges used to believe that surgeons needed to be protected from this threat. Lord Denning, as a supporter of the medical profession, in the case of *Hatcher v Black *[28] described negligence as a "dagger in the doctor's back". It can be always argued that health professionals are human and mistakes and poor practice are unavoidable. Recently, there has been marked increase in the compensation claims against NHS hospital and surgeons. The old belief that surgeons should not be hold liable for their mistakes has now changed [29].

Courts cannot decide whether a surgeon was negligent without expert evidence of "accepted surgical practice" [30]. In any profession, especially in surgery, genuine differences of opinion may arise. It can be found that both sides of debate advance medical reasons for their respective judgment. Also, if deviation from accepted clinical practice is considered to be negligence; this might reflect badly on the advances in all the surgical fields; this suggests that clinicians will not be able to apply an original technique or surgical procedure without facing suspension and a case of negligence. Lord Clyde in *Hunter v Hanley *said that "such thing could be disastrous and severely affect the progress in medical science" [31].

The Compensation Act 2006 was passed as something of a knee-jerk reaction to much publicised claims of a "compensation culture". Section 1 of the Act states that "a court considering a claim in negligence or breach of statutory duty may, in determining whether the defendant should have taken particular steps to meet a standard of care (whether by taking precautions against a risk or otherwise), have regard to whether a requirement to take those steps might- (a) prevent a desirable activity from being taken at all, to a particular extent or in a particular way, or (2) discourage persons from undertaking functions in connection with a desirable activity". It is unlikely for this act to have any effect on the clinical negligence claims, unless judges decided to accept the claims about defensive medicine and surgery, and determine that allowing physicians and surgeons to be sued damages national healthcare [32].

There has been a recent move to the use of guidelines and deviation from them as a proof of negligence [33]. This has been promoted by changes to "The Civil Procedure" [34] which promotes expedience and the use of guidelines even if in the changed rules for disclosure. Unfortunately, as yet many of the guidelines produced by agencies such as NICE have been shown to be flawed even to the extent that they could not be implemented locally because of lack of resources or even patient applicability. NICE has recently been forced to publically apologise for its imposed health inequality for cancer suffers. Even so the House of Lords in "Bland" [35] and The Court of Appeal in "Burke" [36] regarded guidelines as the extension of the Bolam Principle. Several guidelines have been radically changed because of bias of information selection and committee makeup, with each deviation leading to a new raft of negligence claims. The Bolitho test of logic is preferable in treatment selection even if somewhat arbitrary; this is a further move away from the Bolam Principle. It is believed that guidelines will prevent doctors from implementing professional judgment when dealing with their patients; [37] in the field of surgery, every patient should be treated on their own merit.

An uncertainty is not made more certain by the imposition of an arbitrary logic; similarly a procedure that is subject to variation is difficult to test against general guidelines which are meant to be adapted to the individual circumstances of the patient.

The question of professional negligence is problematic because, to a certain degree, each surgical discipline sets its own standards and may to that extent be considered "self-regulating". It is difficult to strike a balance in law between the interests of the professionals and those who rely on them. Courts believe that Bolitho, although welcomed, is being used mainly in a "back-up" position [38].

A situation may arise whereby a surgeon has performed many procedures but has through a fortunate chance not had occasion to have a patient who has experienced a specific complication from these procedures. Would the surgeon be correct in quoting their own quite valid risk figures and percentages without quoting the chance complication that none of the patients treated has experienced but which on average may occur for another surgeon. Eventually the complication may happen but how does a wise surgeon inform the patient in a meaningful way?

In summary, healthcare at best is empirically imprecise and Bolam provides an ideal compromise to allow for this uncertainty. Unfortunately attempts to regulate surgical practice by 'outside agencies' using an idealistic paradigm has led to the 'Bolitho view' and further judgments which although more legally simplistic to apply is based on constructs of logic and the vagaries of the *vox populare *that may not withstand the test to time. Unfortunately, until the occasion of the reasonable and unbiased expert witness, many arguments which suggest logic can be critically but subtly flawed, however to rely upon this "expert" evidence may be fallacious and for a court to decide upon two contrary logical arguments may deviate even further from the truth and eventually justice.

## Competing interests

The authors declare that they have no competing interests.

## Authors' contributions

All authors contributed to conception and design, carried out the literature research, manuscript preparation and manuscript review.

All authors read and approved the final manuscript.
